# Effects of Silicon and Silicon-Based Nanoparticles on Rhizosphere Microbiome, Plant Stress and Growth

**DOI:** 10.3390/biology10080791

**Published:** 2021-08-17

**Authors:** Vishnu D. Rajput, Tatiana Minkina, Morteza Feizi, Arpna Kumari, Masudulla Khan, Saglara Mandzhieva, Svetlana Sushkova, Hassan El-Ramady, Krishan K. Verma, Abhishek Singh, Eric D. van Hullebusch, Rupesh Kumar Singh, Hanuman Singh Jatav, Ravish Choudhary

**Affiliations:** 1 Academy of Biology and Biotechnology, Southern Federal University, Rostov-on-Don 344090, Russia; minkina@sfedu.ru (T.M.); arpnabot.rsh@gndu.ac.in (A.K.); msaglara@sfedu.ru (S.M.); snsushkova@sfedu.ru (S.S.); 2 Department of Soil Science, University of Kurdistan, Sanandaj 66177-15175, Iran; morteza.faizi@gmail.com; 3 School of Life and Basic Sciences, SIILAS, Jaipur National University, Jaipur 302017, India; masudkhann@gmail.com; 4 Soil and Water Department, Faculty of Agriculture, Kafrelsheikh University, Kafr El-Sheikh 33516, Egypt; hassan.elramady@agr.kfs.edu.eg; 5 Guangxi Academy of Agricultural Sciences, Nanning 30007, China; wsb@gxaas.net; 6 Department of Agricultural Biotechnology, Sardar Vallabhbhai Patel University of Agriculture and Technology, Meerut 250110, India; intmsc.abhi@gmail.com; 7 CNRS, Institut de Physique du Globe de Paris, Université de Paris, F-75005 Paris, France; vanhullebusch@ipgp.fr; 8 Centro de Química de Vila Real, Universidade de Trás-os-Montes e Alto Douro, Quinta de Prados, 5000-801 Vila Real, Portugal; rupesh@utad.pt; 9 Soil Science and Agricultural Chemistry, Sri Karan Narendra Agriculture University, Jaipur 303329, India; hsjatav.soils@sknau.ac.in; 10 Division of Seed Science and Technology, ICAR-Indian Agricultural Research Institute, New Delhi 110012, India; ravish@iari.res.in

**Keywords:** silicon, Si-NPs, rhizosphere, microbes, soil properties, abiotic stressors

## Abstract

**Simple Summary:**

Abiotic and biotic stresses are a major challenge for agricultural production. To deal with stressed conditions, many techniques, including the use of nanoparticles (NPs), could be considered to mitigate the adversities mediated by these stresses. The application of silicon (Si) and Si-NPs has emerged as a common agronomic technique as it is regarded as a sustainable option. Because of their innumerable benefits, the usage of Si and Si-NPs has attracted a great deal of interest. As a result, their application has been found to minimize the detrimental effects of various stressors by modifying morpho-physiological indices in plants and rhizospheric microbiome characteristics.

**Abstract:**

Silicon (Si) is considered a non-essential element similar to cadmium, arsenic, lead, etc., for plants, yet Si is beneficial to plant growth, so it is also referred to as a quasi-essential element (similar to aluminum, cobalt, sodium and selenium). An element is considered quasi-essential if it is not required by plants but its absence results in significant negative consequences or anomalies in plant growth, reproduction and development. Si is reported to reduce the negative impacts of different stresses in plants. The significant accumulation of Si on the plant tissue surface is primarily responsible for these positive influences in plants, such as increasing antioxidant activity while reducing soil pollutant absorption. Because of these advantageous properties, the application of Si-based nanoparticles (Si-NPs) in agricultural and food production has received a great deal of interest. Furthermore, conventional Si fertilizers are reported to have low bioavailability; therefore, the development and implementation of nano-Si fertilizers with high bioavailability could be crucial for viable agricultural production. Thus, in this context, the objectives of this review are to summarize the effects of both Si and Si-NPs on soil microbes, soil properties, plant growth and various plant pathogens and diseases. Si-NPs and Si are reported to change the microbial colonies and biomass, could influence rhizospheric microbes and biomass content and are able to improve soil fertility.

## 1. Introduction

Silicon (Si) is not regarded as a necessary element for plants; however, some recent studies reported this element to be beneficial for plant growth. Si is one of the most abundant elements in the Earth’s crust and around 70% of soil mass is made up of Si [1,2,3]. Exposure to Si imparts uncountable beneficial effects on various plants, especially in gramineous and cyperaceous plants [4,5]. In addition, it could alleviate the detrimental consequences of biotic and abiotic stresses that directly or indirectly increase the plants’ resistance to external adversities. For example, it promotes the elongation of roots and alleviates salt stress by reducing NaCl accumulation [2,6]. Si plays a crucial role in several physiological and metabolic processes in plants [7]. For example, in a study, positive effects (enhanced seed germination and chlorophyll content) of Si-based nanoparticles (Si-NPs) on *Zea mays* were observed [8]. Exogenous treatment with Si-NPs reduced salt stress in *Glycine max* by increasing the antioxidant activities, K^+^ concentration and non-enzymatic components, and decreasing lipid peroxidation, reactive oxygen species (ROS) generation and Na^+^ intracellular concentration [9].

Recently, Si-NPs have been documented as a novel Si source that can be used to enhance plant resistance under unfavorable environmental conditions. However, the shape, size and other characteristics of Si-NPs are reported to impact directly or indirectly the responses of plants to Si-NP application [10]. Regarding the efficacy of Si-NPs, it is observed that the soil-applied were more effective than foliar-applied Si-NPs [11]. Si-NP treatment improved the growth and oil content in *Cymbopogon citratus* [12]. It enhanced the growth of *Avena sativa* and led to lignification in plant tissues [13]. It was reported that a nano-silica fertilizer improved the leaf area index, net assimilation rate, relative growth rate and yield of *G. max* [14]. Seed priming and seed soaking of *Helianthus annuus* in Si-NPs improved seedlings’ shoot and root length, biomass and vigor index [15].

Seed germination and seedling growth of *Agropyron elongatum* have been found to be improved by silicon dioxide nanoparticles (SiO_2_-NPs) [16]. Nano-SiO_2_-based fertilizers are determined to be beneficial for crops as they minimize fertilizer loss such as nitrogen and phosphorus by controlled release [17]. The application of SiO_2_-NPs could improve the photosynthetic pigments and increase the photosynthetic rate [2,3,18]. It also improved seed germination in *Solanum lycopersicum*; the net photosynthetic rate, photochemical efficiency, photosystem II (PSII) activity, electron transport rate, carbonic anhydrase activity, photochemical quenching, stomatal conductance and transpiration rate in *Indocalamus barbatus* and *Cucurbita pepo* [19,20]; and it also increased the growth, chlorophyll and carotenoid contents of *Solanum tuberosum* tubers [21]. Plants absorb Si in the form of mono-silicic acid and it accumulates in different tissues [18,22], and its deposition may occur in the leaf, stem and vascular tissues [1,23] and cuticles of plants [24]. In plants, there are Si transporters in the cell membrane (low silicon; Lsi1, Lsi2) and these transporters function as influx and efflux transporters [1,25,26].

After a review of the literature, some authors observed that Si is beneficial in controlling a variety of plant diseases by triggering the host defense system [27,28]. The effects of nano-silica (i.e., synthesized using *O. sativa* husk) and conventional Si on the bacterial population and seed germination of *Z. mays*, as well as the soil properties, have been evaluated [29]. Soil treated with sodium silicate hindered the colonization of plant-growth-promoting rhizobacteria, whereas the application of nano-silica enhanced the bacterial population. As a result, Si could boost plant resistance to bacteria, fungus, nematodes and viruses [30]. Si is reported to modulate the signaling systems that normalize the expression of defense genes related to proteins, the structural modification of cell walls, antimicrobial compound synthesis, hypersensitivity responses and hormone synthesis [31]. It was found that Si stimulated resistance in *Solanum lycopersicum* against *Ralstonia solanacearum* by upregulating defense gene expression [32]. The application of Si changed 26 proteins in *S. lycopersicum* inoculated with *R. solanacearum*, and it also changed the protein level in the host plants [33]. It was also noticed that the application of Si reduced the infection of *Magnaporthe oryzae* in *O. sativa* [34]. The application of SiO_2_-NPs was observed in the management of a *Meloidogyne incognita*, *Pectobacterium betavasculorum* and *Rhizoctonia solani*-mediated disease complex in *Beta vulgaris* L.; thus, its potential to reduce disease severity has been revealed [30]. Therefore, these research outcomes have shown that Si could reduce pathogen invasion in plants [35].

Therefore, this review article aims to assess the impacts of Si and Si-NPs on soil microbes, soil properties and their effect on plant growth and diseases.

## 2. Source of Si and Si-NPs and Their Uptake

Si is an element with a Van der Waals radius of 210 pm that exists as different forms in the environment, while Si nanoparticles are synthesized particles that are smaller than 100 nm with special properties. The unique characteristics of Si nanoparticles have made them effective reagents in agricultural applications. Unlike bulk silicon, a very dull material, ultrasmall silicon nanoparticles are extremely efficient at ameliorating soil properties [36]. Si could be found in the soil solution in a variety of forms, including monomeric (H_4_SiO_4_; monosilicic acid), oligomeric and polysilicic acid. The monomeric form is readily bioavailable for plants [25]. Plants are recorded to accumulate Si up to a significant amount and earlier studies revealed that there are three different modes of Si uptake, namely active, passive and rejective [37]. In the active mode, plants absorb Si at a faster rate than water, resulting in a lower Si concentration in the uptake solution; yet, in passive mode, Si absorption by plants is similar to water uptake. Thus, in passive uptake, there are no discernible changes in the concentration of Si in the uptake solution. Plants that uptake using a rejective mode prefer to exclude Si, as seen by the increasing concentration of Si in the uptake solution [38]. However, there is still great room for further investigation to depict the mechanisms involved in the different uptake modes of Si.

**Figure 1:**
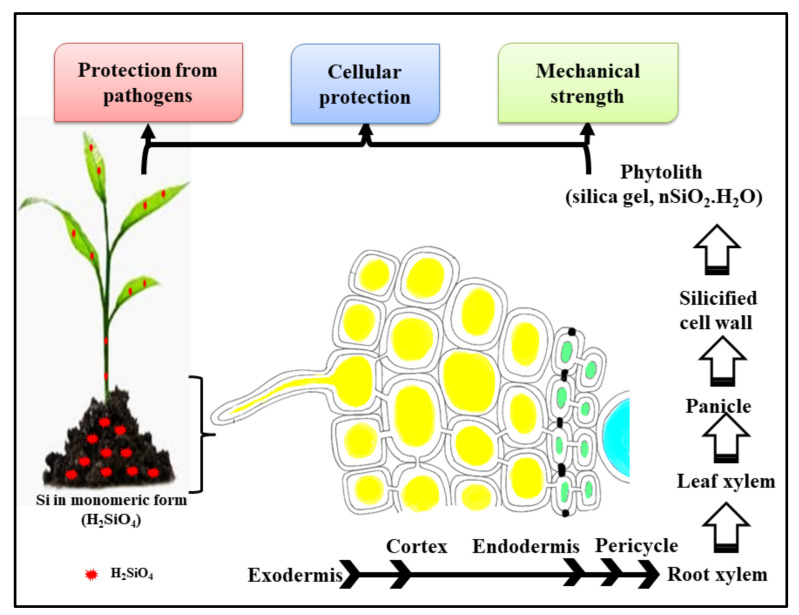
Diagrammatic presentation of silicon transport in plants.

In the roots, after the uptake of Si-NPs, their transport to other aerial parts is reported by three routes viz., cell wall pores, the apoplastic pathway and the symplastic pathway (Figure 1). The Si-NPs are recorded to travel either intracellularly or extracellularly before they enter the xylem, according to the existing literature [39]. It was noted that the critical value of soil-available Si content for *O. sativa* is 300 mg SiO_2_ kg^−1^.

Based on Si uptake accumulation, plants are categorized as high, intermediate and low Si accumulators. It is assumed that in high Si accumulators, the amount of H_4_SiO_4_ taken up by active mechanisms is greater than concentrations taken up by mass flow due to the high density of Si transporters in roots and shoots expediting H_4_SiO_4_ movement through root cell membranes (Figure 1) [37,40]. The transport of Si is a multi-step process and, from roots to shoots, silicic acid crosses the plasma membrane at biological pH. The first Si transporter (Lsi1) was discovered in *O. sativa* and belongs to the Nod26-like major intrinsic protein subfamily [41]. According to the findings of previous studies, the site of Si uptake is in the mature regions of the roots rather than the root tips due to the higher expression of Lsi1 genes than the apical region. Further, the expression of Lsi1 in rice at various growth stages was found to be transiently increased around the heading stage. It has also been shown that the maximal amount of Si was taken up during the reproductive stage from panicle initiation to heading in *O. sativa* [42].

As Lsi1 are responsible for the influx of silicic acid from external media to cells, similarly, for the efflux of Si, there are Lsi2 transporters. The mechanism of Si transport mediated by Lsi2 is an energy-dependent active process, i.e., led by the proton gradient. Lsi2 is expressed in the roots, similarly to Lsi1 [43]. Thus, the expression of Lsi1 and Lsi2 is reported to be regulated in a similar manner. Both Lsi1 and Lsi2 are localized at the exodermis and the endodermis cells of the roots. The main difference is in their localization; Lsi1 is localized on the distal side, while Lsi2 is localized on the proximal side of the exodermis and the endodermis cells [44]. Si influx has also been identified in other plants, e.g., *Z. may*s (ZmLsi1) and *Hordeum vulgare* (HvLsi1), whilst very few efflux transporters in other plants have been identified so far [45].

In the soil solution, certain dissolved silicon acid forms organic and inorganic compound complexes. Silcretes are a form of derived soil that contains a significant amount of Si. In petrocalcic horizons, the Si amount is much smaller than in silcrete (8%), while it is significantly lower in minerals of certain heavily weathered oxisols such as bauxites and ferricretes [46]. Most of the soils are rich in Si; however, some soils are poor, especially the plant-available type of Si [47]. The oxisols and ultisols are heavily weathered, leached, acidic and also display poor base saturation [48]. Meanwhile, the histosols possess a great deal of organic matter and very low mineral content [49]. Furthermore, soils with a high proportion of quartz sand and those that have been subjected to long-term crop productivity have low plant-available Si [46,50].

Among these, silica is listed as one of the crystalline types of Si in the solid phase fraction [51]. The primary and secondary crystalline silicates, which are abundant in mineral soils formed from rocks and sediments, were previously the only crystalline types [52]. Quartz and disordered silica make up the majority of silica products. The Si fractions in the solid phase also include amorphous, poorly crystalline and microcrystalline shapes [52,53]. The liquid and adsorbed phases of Si are identical, with the exception that the liquid phase components are dissolved in the soil solution, while the adsorbed phase components are retained on soil particles as well as on Fe and Al oxides/hydroxides.

Si content and its abundance in soils are closely dependent on processes of soil formation and consequently on the soil type. Except for organic soils (histosols), most mineral soils are made up of sands (mostly SiO_2_), different forms of primary crystalline (e.g., olivine, augite, hornblende, quartz, feldspars-orthoclase, plagioclase, albite, and mica) and secondary minerals of silicate including clay minerals such as illite, vermiculite, montmorillonite, chlorite and kaolinite. These silicate compounds are, in most cases, not very soluble and are biogeochemically inert. In soil, polymerized silicic acid is only partly water-soluble, while H_4_SiO_4_ is the water-soluble type of Si. Water-soluble Si can be adsorbed onto the surfaces of inorganic, organic and organic–inorganic complexes in soil [25,47,52,54].

## 3. Impacts of Si-NPs on Soil Properties

Natural nano-sized materials found in soil include Si, Al, K, Na, Ca, Fe, Ba, Sr, Rb, as well as silicates, carbonates, sulphates, oxides, hydroxides and phosphates [55]. The soil properties viz., soil texture, pH, soil salinity (EC), soil organic matter, cation exchange capacity (CEC), etc., control the fate and behavior of any element in the soil rhizosphere [56]. Moreover, the rhizosphere itself can also regulate the movement of the elements. Thus, cultivated plants also might impact the uptake of the element in the soil, even when the elements are in nano-form (Table 1).

Three main types of Si in the solid state in the soils are amorphous (i.e., poorly crystalline), microcrystalline and crystalline. The crystalline types of Si are primarily used as silicates and silica materials (primary and secondary), and they account for the majority of Si in the solid phase. The primary mineral-bearing silicates inherent in soils are contained in sand and silt particles, while the secondary silicates are found in clay particles formed by pedogenic processes involving phyllosilicates and Al-Fe oxides/hydroxides [52]. The Si exists as poorly crystalline and microcrystalline types, such as short-range ordered silicates, chalcedony and secondary quartz [52].

Amorphous forms are biogenic and lithogenic and are available at quantities of up to 30 mg g^−1^ total soil. The biogenic types, which consist of plant residues and the remains of microorganisms, are called biogenic opals. These biogenic opals are formed when the soluble Si in the soil is supersaturated [57]. Plants accumulate Si as phytoliths in their leaves, culms and stems, while microorganisms contribute as microbial and protozoic Si [52,58]. The solubility of various Si types in the solid stage significantly affects the concentration of their soil solution. The solubility of minerals containing silica depends on the density and range of the silica tetrahedrals [57,59]. Further, amorphous silica is anticipated to contribute to higher solubility than quartz. However, quartz is extremely stable and thermodynamically resistant to weathering; its solubility ranges from 0.10 to 0.25 mM Si [57]. Thus, if the quartz is abundant in residual compounds, then its contribution to Si in soil solution is negligible [60].

The solubility of both amorphous and crystalline silica is documented to be nearly constant at around pH 2.0 and 8.5. However, their solubility quickly increases at pH 9.0 as the concentration of H_4_SiO_4_ decreases in the soil solution due to the dissociation of H_4_SiO_4_ into H_3_SiO_4_^−^ and H^+^ at pH 9.0 [61]. This allows the crystalline and amorphous silica to dissolve in order to replenish or buffer the decreased concentration of H_4_SiO_4_ in the soil solution [47]. The plant-available forms of Si present in soil range from 10 to 100 mg kg^−1^. In soil, less than 20 mg kg^−1^ Si is considered a Si deficit and the amendment of Si is recommended [54].

In addition, Si-NPs can inhibit the leaching and movement of heavy metals in soil. For instance, it was confirmed that the application of Si-NPs subsequently improves the stable concentrations of Cu, Zn and Ti oxides [77]. To provide detailed insights, in Table 1, we summarize the research outcomes of recent studies that demonstrate the influences of soil properties in cultivated plants and their association with the application of Si-NPs.

## 4. Effects of Si-NPs on the Rhizospheric Microbiome

Soil is a reservoir of water and nutrients for plants and is, therefore, indispensable for the plants’ normal functional processes. The surrounding area of the roots of plants is known as the rhizosphere. In the rhizosphere complex, biotic and abiotic relations exist. Thus, the rhizospheric microbiome comprises many microbial organisms, including archaea, viruses, fungus, bacteria as well as eukaryotic microorganisms, which are directly linked to the plant roots in a compact region of soil. It is also documented that approximately up to 10^11^ microbial cells g^−1^ of the root are present in this rhizospheric unit, accounting for more than 30,000 prokaryotes [78]. Moreover, the rhizospheric component of the soil is characterized as a zone that is influenced by exudates and roots’ secretions, which are vital for plant growth and health along with the microbial community [79]. The release of a variety of soil metabolites viz., organic acids, inorganic acids, siderophores, sugars, vitamins, amino acids, purines, nucleosides, polysaccharide mucilage, etc., is reported by different researchers. Thus, this subset of soil microbial diversity is reported to be sensitive to numerous chemical substances, including NP application and other physicochemical changes in the rhizosphere that subsequently favor the selective enrichment of certain microbial communities over others [80].

**Figure 2:**
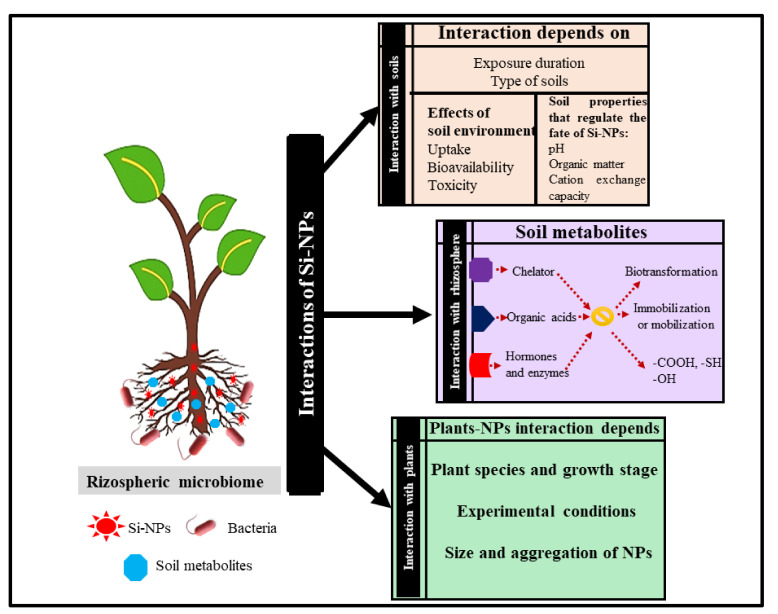
Schematic representation of interactions of Si-NPs with rhizospheric microbiome.

The microflora present in this region tends to be pathogenic as well as beneficial [81]. The beneficial rhizospheric microorganisms play a pivotal role in the immobilization/cycling of nutrients along with the detoxification or degradation of pollutants, which results in improved soil health (Figure 2).

The rhizosphere contains advantageous microorganisms such as phosphate-solubilizing bacteria (PSB) and nitrogen-fixing microbes [82]. These microbes are plant growth promoters and thus can exert modulatory impacts on the biological and chemical properties of soils. Silicate-solubilizing bacteria (SSB) are also present in soil and they could convert insoluble silicates into soluble Si and alleviate Si content in the soil. Thus, the rhizosphere plays a vital role in the maintenance of soil properties and plant health [83]. In this context, treatment with Si-NPs and conventional Si is found to enhance the microbial biomass in the soils and the availability of Si to plants. Further, Si-NPs play an important role in influencing microbial biota and soil nutrient content; hence, their application is recorded to promote the growth of crops [84]. In this study, the PSB population (3.8 × 10^4^ CFU g^−1^) was observed to increase after Si-NP treatment, whilst there was no impact on the population of SSB. Likewise, in a study, it was reported that the application of Si-NPs had a significant impact on soil nutrient content and microbial biota and thus improved *Z. mays* growth [11].

Nitrogen-fixing bacteria have a high population among the Si amendment soil. The foliar application of SiO_2_-NPs increased the bacterial communities of *Paenibacillus* and *Rhodobacteraceae* [85], and also improved *Chaetomium* fungal genera in the rhizosphere. Moreover, in this study, the comparative profusion of the genus *Paenibacillus* in the phylum *Firmicutes* was approximately 16% higher in the soil with NPs than in the control. The genus of *Paenibacillus* includes plant growth-promoting bacteria, which encourage plant growth via different mechanisms, such as nutrient solubility, biological nitrogen-fixing, induction of systemic resistance and plant growth regulators and organic acid production [86]. These microbes are vital for the nitrogen and carbon cycles. Si- and SiO_2_-NP uptake and their impact on soil microbial colonies require a thorough and deep investigation. Thus, treatment with Si and Si-NPs can be beneficial due to their direct or indirect influences on the economic productivity of plants.

Soil metabolites are important intermediates in many soils’ productivity and fertility processes. Thus, shifts in the root exudate will affect the plants’ health and growth levels or composition by attenuating soil fertility. In this context, soil metabolomics provides a potential method for soil characterization and the evaluation of the soil microbial community’s metabolic status, as shown in a high-performance study on small molecular organic compounds [87]. In another study, it was indicated that the rhizosphere metabolite profile was significantly influenced following the foliar exposure of SiO_2_-NPs [85]. The considerable increase in the relative profusion of numerous metabolites, including sugar and sugar alcohol, fatty acids and small organic acids, confirmed the influences of NPs on carbon and nitrogen pools in the rhizosphere. Others noted the impact of some NPs such as Si-NPs and their results indicated that oversaturation of these NPs reduced dehydrogenase and urease activity as well as bacterial and archaeal amoA gene abundance in soil [81]; it was confirmed that a mixture of Cu, Ag and Si decreased C and N biomass and changed the microbial community structure in soil [88].

## 5. Effects of Si-NPs on Plant Growth and Development

Nanotechnology is reported as a cutting-edge technique that has been proven to be more efficient for phytoremediation along with its application in stress mitigation [2,89]. Si-NPs may improve crop yield by influencing nutrient availability in rhizospheric soil and absorption by the plants. Si-NPs improve the nutrient bioavailability in plants, thus acting as a primary reason for the increased plant growth following NP application [6,90]. Due to the importance of Si in plants, similar to other essential macronutrients, as an agricultural nutrient, scientists have focused on applying Si-NPs in the soil in order to improve plant growth. Moreover, Si deficiency has been linked to nutritional imbalances, resulting in poor growth [59].

In a recent study, it was found that Si-NP priming of different seeds viz., *T. aestivum*, *Pisum sativum* and *Brassica* improved the parameters of seed germination and seedling growth [91]. Several reports have revealed that Si-NPs act as a fertilizer [3,20,92]. In addition, treatment with Si-NPs reduced the negative effects of salt stress on vegetative growth and soil relative water content, resulting in a considerable improvement in plant height, fresh and dry weights, total yield and seed quality. The various indices related to *Z. mays* plants’ physiology and anatomy were considerably altered after exposure to Si-NPs at 20–40 nm with a high surface area.

The impact of Si-NPs on plants is affected by various factors, i.e., the size, shape, application phase and biomechanical and physical properties [10]. According to some reports, Si-NPs can communicate directly with plants, altering their morphological behavior and physiological activity in different ways [20]. Si-NPs, on the other hand, have been shown in numerous experiments to be harmful to plants [54,93]. Therefore, some important studies involving plants and Si-NPs are shown in Table 2 to provide a better understanding.

Si-NPs can also act as a strengthening substance that is responsible for improving disease resistance by preventing fungal, bacterial and nematode infections. Si-NPs can also reduce the transpiration rate of the plant, rendering it more resistant to limited water supply (drought), high temperature and humidity [18,46,93,102]. Except for a few scientific papers indicating that Si-NPs have a negative impact on plant performance, most of the studies found Si-NPs to be beneficial or ineffectual for plants by either promoting plant growth or having no impact [7,11,102].

Plants produce naturally mineralized NPs for proper growth and development when subjected to different stresses [93]. The use of Si-NPs ensures better plant performance and yield during unfavorable environmental conditions. The high surface-to-volume ratio of Si-NPs is reported to increase their reactivity and biochemical activities, which is responsible for their favorable effects on plants [89,103].

## 6. Role of Si and Si-NPs in Abiotic Stress Tolerance in Plants

Drought, heat, salinity, heavy metals and salt contamination of soil are all critical environmental stressors that severely affect the productivity and quality of agricultural species around the world. Plants’ physiology, morphology and biochemistry are altered by abiotic stresses, resulting in reduced growth and economic output [18,93,104]. Thus, Si-NPs have been reported to serve indispensable roles by mitigating different abiotic stress-induced consequences [4,70,105,106]. For example, metal toxicity can be minimized and plant growth can be improved by the amendment of Si-rich materials in soils [18,93]. Si-NPs can significantly decrease the heavy metal content in plants. In a study, it was reported that the application of Si-NPs diminished the content of Pb in the different tissues of *Brassica chinensis* L. as compared to the control [85]. These results suggest that the exogenous application of Si-NPs can minimize heavy metal uptake in plants [97,107]. 

Cadmium is one of the most hazardous toxic metals on the planet [108]. It inhibits plant growth, photosynthesis and yield [2,109]. Si-NP treatment of Cd-stressed plants increased the plant growth and biomass [110]. Moreover, it was concluded that Si-NP treatment of the soil could promote plant growth indicators and photosynthesis while lowering Cd levels in plants’ tissues, particularly in grains that are or are not experiencing drought stress [30]. Moreover, Si-NPs lessened oxidative stress, as evidenced by decreased H_2_O_2_ generation, electrolyte leakage and malondialdehyde levels, as well as increasing superoxide dismutase and peroxidase activity. Further, Si treatment protects cell membranes from injury [111]. The application of Si-NPs reduced reactive oxygen species (ROS) levels and boosted antioxidative defense components in Cd-stressed plants [68].

The foliar application of Si-NPs significantly reduced the accumulation of Pb in the leaves of *O. sativa* [110]. Treatment with Si-NPs in soil was also used as a unique strategy to alleviate Al phytotoxicity in acidic soils, and a thorough view of the cellular and biochemical mechanisms behind this mitigation process was provided. Arsenic is a metalloid that is toxic to plants and adversely affects plant growth [112,113]. Si-NP treatment reduced As stress-mediated vulnerabilities in *O. sativa* [114]. Si-NPs were used to prevent damage and restore the photosynthetic mechanism. Si-NPs also enhanced the activities of antioxidant enzymes to counter ROS generation to reestablish cellular homeostasis [112].

Here, we consider the consequences of chromium stress-induced responses in plants. Generally, Cr is reported to accumulate in plants, causing changes in photosynthetic activity, nutrient uptake and plant development [115]. Si-NPs are reported to improve *P. sativum* seedlings’ growth under Cr stress. It was found that the application of Si-NPs to Cr-stressed plants ameliorated Cr-induced phytotoxicity symptoms, i.e., pigment content, chlorophyll fluorescence, proteins level and nutrient status, resulting in improved growth [112]. The reduction in Cr accumulation in plant organs is followed by an improvement in physiological indices. The ability of Si to upregulate the expression of osNAC proteins, which are responsible for the upregulation of genes involved in stress tolerance, proline synthesis, soluble sugar biosynthesis and redox homeostasis, could be a reflection of Si-NPs’ increased stress tolerance. The significant roles of Si-NPs in mitigating metal toxicity in maize (*Zea mays* L.) plants were recorded, which resulted in enhanced photosynthesis responses, reduced oxidative stress, i.e., ROS, H_2_O_2_ and malondialdehyde (MDA) content, and maintained antioxidative defense mechanisms. The application of Si-NPs decreased MDA content during metal toxicity and positively improved cell wall breadth in the epidermis of roots, while also downregulating metal ion absorption and the accumulation rate [99]. Si-NPs are easily absorbed by plants as compared to inorganic Si and protect against excess metal ion toxicity in crop plants [116].

Si-NPs significantly boost the germination and vigor index in *Cucurbita* plants during saline stress conditions [20]. Si is reported to balance the homeostasis of ions and mitigate abiotic stresses. Si-NPs provided a favorable environment for seed germination under salinity stress [19]. The enhancement of growth indices might reflect the photosynthetic functions of Si-NPs, which is needed for photosynthetic leaf gas exchange and the assimilation of nitrate [104,117,118]. However, Si-NPs have been implicated in the synthesis of proteins and amino acids and in nutrient uptake; the strength and rigidity of plants are improved through Si-NP deposition in different plant organs [43]. Si-NPs increased the morphological and photosynthetic traits of plants via enhancing organic compound production, i.e., proteins, pigments and phenols, relative to bulk particles [4,12]. Under unfavorable environmental conditions, plants synthesize compatible solutes, i.e., glycine betaine and proline, to maintain the osmotic potential within plant cells. The level of proline is enhanced with Si-NPs. Proline is a universal osmoprotectant, acts as an antioxidative and energy source [119] and regulates the expression of genes, leading to osmotic adjustment [19,120].

Si-NPs have a potential role in *S. lycopersicum* germination (%), time, index, vigor index, fresh and air-dried biomass of plants [20]. Under saline conditions, the amendment of nutrient media with Si-NPs enhanced the seed germination and seedling early growth of *Lens culinaris* [15]. Si-NPs decreased the effects of saline toxicity in *Ocimum basilicum* and enhanced the fresh and dry mass of plant organs, leaf chlorophyll index and proline level [46]. In another study, Si-NPs were used to enhance the photosynthetic capacity and mitigate the seed germination and plant growth inhibition caused by salinity in *S. lycopersicum* plants [121]. Salt-stressed genes, i.e., AREB, TAS14, NCED3 and CRK1, were found to increase their expression in *S. lycopersicum* subjected to saline conditions with the application of Si-NPs, while RBOH1, APX2, MAPK2, ERF5, MAPK3 and DDF2 genes were noted to be downregulated [121]. Si triggered modifications in plant cell metabolism, a decline in heavy metal uptake by roots and the exudation of specific chemicals such as organic acids and phenols [122,123].

In addition, plants can use Si-NPs as a carrier of important macronutrients (N, P and K) and as a slow-release Si supplement to help them cope with salt stress [124]. Thus, in this context, these materials can be considered growth-promoting agents. Si supplementation is considered to inhibit salinity in plants, so Si-NPs have been used to improve salinity tolerance in plants [124]. Moreover, for regulated release, NPK fertilizers and Si-NPs should be carried inside the core of controlled-release fertilizers, which can be coated with chitosan as the first semipermeable coating and sodium alginate and kaolin as the outermost superabsorbent coating [125]. These artificial beds could slowly disperse nutrients, allowing plants to hold enormous amounts of water, manage salinity and thrive in drought circumstances [124].

## 7. Role of Si and Si-NPs in Plant Biotic Stress Management

The application of Si decreases biotic stress severity in many plants. Si has the potential to help plants to avoid pathogen penetration by forming physical barriers and suppressing pathogen colonization by boosting systemic acquired resistance. Moreover, Si protects plants by strengthening the host plant’s cuticle and cell walls, as well as producing silicate papillae, which impede the spread of pathogenic structures. Si accumulation in the cell wall of the host plant to form a double layer of silica makes the penetration of pathogens difficult [126]. Si accumulation can lead to the deposition of a double layer beneath the cuticle, preventing pathogen penetration and lowering disease incidence.

Si may play an active prophylactic role in plants. Phenolic-like compounds were found at a high level in Si-treated plants upon infection with pathogens [127]. Si binds with pectins, polyphenols and hemicellulose in cell walls and improves the mechanical strength of the plant cell wall [112,128]. Si provides rigidity to the cell wall. Si treatment in rice induced resistance against pathogens and reduced blast disease [126]. In *T. aestivum* leaves, Si application inhibited the hyphae penetration of *Pyricularia oryzae*, while, in the absence of Si, fungal hyphae penetrated successfully [105]. Similarly, Si treatment in *O. sativa* reduced the infection and leaf lesions caused by *Rhizoctonia solani* and *Pyricularia grisea* [129,130]. Moreover, *P. oryzae* penetration in *O. sativa* tissues decreased after Si treatment and it was suggested that the presence of the Si layer helped in blocking or delaying pathogen penetration [131]. Si reduced sheath blight disease of *O. sativa* in treated plants [129]. After treatment with Si, the number of *Podosphaera fuliginea* colonies was reduced by 43–94% in *Cucumis sativus* [132]. Si treatment reduced the blast lesion length in *O. sativa* by 40–80% [130].

Si-NPs exhibit potent antibacterial properties against a variety of plant diseases and are thought to help in the regulation of soil N levels [133]. Si-NPs could hamper the growth of pathogenic fungus (*Fusarium oxysporum f.* sp*. niveum*) [134] and may reduce the growth of plant parasitic nematodes (*Meloidogyne incognita*), bacteria (*Pectobacterium betavasculorum*) and fungus (*Rhizoctonia solani*) [30]. In a study, Si-NP treatment improved the growth of *P. sativum*, but the highest growth was recorded when SiO_2_-NPs and N-fixing bacteria (*Rhizobium leguminosarum*) were applied together [135]. SiO_2_-NP treatment reduced the bacterial blight disease complex of *P. sativum* caused by a plant parasitic nematode (*Meloidogyne incognita*) and bacterium (*Pseudomonas syringae* pv. *pisi*). Detailed studies of the effects of Si and Si-NPs on plant biotic stress management, especially diseases, are listed in Table 3.

## 8. Molecular Mechanism of Si and Si-NP Uptake and Their Applications in Agriculture

In plants, variations in the accumulation of Si occur. In the bryophytes and pteridophytes, high Si accumulation occurs as compared to the angiosperms. In angiosperm families, the genera of Poaceae and Cyperaceae have been reported to accumulate high amounts of silicon, whilst the Urticaceae, Commelinaceae and Cucurbitaceae families have intermediate silicon accumulation [142,143]. Rice belongs to the Poaceae family and it can accumulate around 10% Si. Hodson et al. [143] performed a meta-analysis that recorded the following order of Si accumulation in various plant groups (from high concentrations to low): liverworts > horsetails > clubmosses > mosses > angiosperms > gymnosperms > ferns. Silicon uptake in plants is attributed to specific transporters.

Si is present in the soil and its uptake and transport in plants depend on the chemical composition and plant roots. Si is taken up by plant roots as monosilicic acid and transported to the shoots. Deposition of Si mostly occurs in leaf epidermal cells, in the outer epidermal cells of inflorescence bracts and in the root endodermis. Due to the transpiration, Si concentrates and polymerizes into colloidal silica gel (SiO_2_·nH_2_O). Uptake of Si in rice plants is faster, indicating the presence of Si transporters across the cell membrane [142]. To understand the molecular mechanism of Si uptake and transport in rice, a mutagenic approach has been used and it was found that the transporter gene Lsi1 plays an important role in Si transport. Shortly after the discovery of Lsi1, a second Si transporter gene, Lsi2, was discovered. Lsi2 has 9–12 transmembrane domains and it is an anion transporter coupled with a proton antiport. The discovery of these transporters has explained the molecular mechanism of Si uptake and transport in rice plants [1,2,142]. In *Z. mays* plants, Si uptake and deposition were mediated by ZmLsi1 and ZmLsi6 genes. ZmLsi1 is found to play a key role in the uptake of Si by roots. ZmLsi6 is located in the parenchyma cells of *Z. mays* leaves and it is responsible for Si unloading in xylem. Homologs of Lsi2 and HvLsi2 genes have also been reported in *H. vulgare* plants [144].

Si and Si-NPs have promising applications in agriculture. Si-NPs have a beneficial impact in the agriculture sector and they may improve yields, leading to increased productivity [145]. Moreover, additional applications of Si-NPs include pesticides, fertilizers and herbicides. Si-NPs could be used to develop NPs. Several studies have shown that nano-silica increases the efficiency and durability of pesticides. Si-NPs could be used for the target-specific delivery of fertilizers and herbicides. Mesoporous silica NPs with a pore size of 2–10 nm served as an efficient delivery vector for boron, urea and nitrogenous fertilizers. Li et al. [146] observed that Si-NPs increased the photostability and sustained the release of an avermectin pesticide.

## 9. Conclusions and Future Perspectives

The benefits of Si to a variety of crops have long been known, implying the importance of Si fertilization as a long-term option for viable agriculture. Agricultural lands with rigorous cultivation systems, particularly those that are naturally low in soluble Si soils, can be modified with Si-rich materials to ensure productivity. Recently, the use of Si in soil fertilization has emerged as a common agronomic practice in several parts of the world. Despite significant advances in Si research and soil science in the development and standardization of multiple processes for the extraction and quantification of various soil Si fractions, their use in soil fertility and nutrient management is limited. The soil interpretation test could be used to assess whether Si-based fertilization is necessary or not, but it does not provide the exact Si concentration required to increase Si availability to the optimal limit, nor does it indicate whether the affected plant will respond to Si fertilization. Thus, in the near future, soil science-based Si research studies are expected to make substantial progress in current soil Si knowledge and provide crop-producing fertilization recommendations.

## Figures and Tables

**Table 1:** The impacts of soil properties on cultivated plants and their links to applied nano-silica.

Details of Si-NPs		Soil Properties				Main Effect	Reference
Nano-Si Dose and Type of Preparation	Size (nm)	Texture	pH	SOM (g kg^−1^)	EC (dS m^−1^)		
Chemical nano-SiO_2_ (1.5 mM mg Si L^−1^)	20–35	Clay loam	7.84	10.3	1.03	Improves growth and oil yield of coriander under drought stress	[62]
Chemical nano-SiO_2_ fertilizer (2%)	80–90	Silty clay	7.99	9.2	1.1	Records the best yield of wheat under water deficit	[63]
Chemical nano-SiO_2_ (60 mg L^−1^)	20	Conditioned soil (data not available)				Mitigates stress in rice by enhancing antioxidant system	[64]
Chemical chitosan-Si-nano-fertilizer (from 0.01 to 0.16%, *w*/*v*)	100	Clay	8.20	13.78	0.56	Enhances maize growth and yield by inducing antioxidant defense system	[65]
Chemical thiol functionalized nano-SiO_2_ (4%)	20	Silt loam	7.93	13.78	NA	Remediates polluted soil from heavy metals and improves growth of lettuce	[66]
Biological Si-NPs (2.5 and 5.0 mmol L^−1^)	38.78	Loamy	7.30	6.88	7.81	Promotes common bean under saline and polluted soil with Pb, Ni and Cd	[67]
Chemical SiO_2_-NPs (0.75, 1.5 and 2.25 mM)	10–20	Sandy loam	7.10	NA	1.20	Mitigates Cd stress by improving antioxidants and growth of summer savory	[68]
Chemical Si-NPs (100–200 mg kg^−1^)	8.3	Clay loam	6.60	7.0	0.70	The 200 mg kg^−1^ nano-Si + PGPB recorded highest level of Si-soluble and exchangeable fractions under water deficit stress	[69]
Chemical SiO_2_-NPs (150–2000 mg kg^−1^)	10	Commercial soil	7.35	NA	NA	Treatment of 500 mg kg^−1^ lowered the content of As and Cd to 70 and 50% under the water regimes in rice shoots	[70]
Chemical SiO_2_-NPs (at 2 mM)	30	Clay loam	7.40	2.80	1.7	Improves maize yield under applied nano-Si in combined with Zn nutrient	[71]
Surface-modified nano-silica (3.0%)	18.0	NA	7.61	16.9	NA	Immobilizes bioavailable As, Pb, Cd, by 85, 97.1, 80.1%, res. in polluted soil	[72]
Chemical nano-Si complex with glycine, glutamine, histidine	10–40	Silty loam	7.02	NA	0.62	Nano-Si enhances growth of feverfew under drought stress at foliar 1.5 or 3 mM	[73]
Chemical Si-NPs (at 1 and 2 mM)	20	Loamy	8.08	8.0	1.11	Improves antioxidants to protect sugar beet plants from water deficiency stress	[74]
Chemical mercapto-functionalized nano-silica (0.2 to 0.1%)	20–30	NA	8.12	19.6	NA	Increases wheat grain yield by 33.5% and soil dehydrogenase by 43.4% under Cd stress	[75]
Chemical nano-SiO_2_ (500 mg kg^−1^)	NA	Sandy loam	7.67	2.54	NA	Enhances remediation by *Erigeron annuus* L. grown in polluted soil by PAH (150 mg kg^−1^)	[76]

Abbreviations: PGPB: plant-growth-promoting bacteria (*Pseudomonas*19 sp.); NA: not available; PAH: phenanthrene.

**Table 2:** Role of silicon nanoparticles in plant growth and development.

Structure of Si-NPs	Crop	Concentration	Adaptive Mechanism	Reference
SiO_2_ (chemical)	*Saccharum officinarum* L.	300 ppm	Improves leaf photosynthetic responses, chlorophyll fluorescence yield, photosynthetic pigments and photosynthetic apparatus (PS II) during chilling stress	[94]
SiO_2_ (chemical)	*Glycine max* L.	100–2000 ppm	Si-NPs increase plant performance and reduce the uptake of Hg in epidermis and pericycle of roots and stems. They enhance photosynthetic content and antioxidant enzyme activities in soybean during exposure to mercury (Hg)	[95]
SiO_2_ (chemical)	*Hordeum vulgare* L.	125–250 ppm	Improves plant development, green pigments, photosynthetic activities, plant osmolyte and metabolite profiles, cellular damage and membrane stability indicators, and antioxidant enzymes are all affected.	[96]
Si-NPs (chemical)	*Oryza sativa* L.	1 mM	Enhances gene expression and transportation of cadmium to vacuoles.	[97]
SiO_2_ (commercial)	*Trigonella foenum*L.	0–2.5 mM	Increases nanoparticle translocation, accumulation, Si uptake, cell wall lignification and the formation of stress-related enzymes during metal toxicity (cadmium)	[98]
Si-NPs (chemical)	*Triticum aestivum*L.	10 µM	Mitigates negative effects of UV radiation on plants	[99]
Mesoporous Si-NPs (chemical)	*Triticum* spp. L., *Lupinus polyphyllus*L.	200–2000 ppm	Nanoparticles upregulate leaf gas exchange responses and growth development performance of plants	[100]
SiO_2_ (commercial)	*Pisum sativum* L.	10 µM	Protects plant seedlings and increases enzymatic activities	[101]

**Table 3:** Effects of Si and Si-NPs on plant biotic stress management.

Nanoparticle Type	Pathogen	Concentration	Effect	Reference
Si-NPs	*Fusarium oxysporum f.* sp*. niveum*	100 mg L^−1^	Enhances biomass and fruit yield in comparison to untreated plants	[134]
SiO_2_-NPs	* Meloidogyne incognita, Pectobacterium betavasculorum * and *Rhizoctonia solani*	100, 200 mg L^−1^	Si-NPs were most effective against test pathogens	[30]
SiO_2_-NPs	* Xanthomonas campestris pv. carotae, Pectobacterium carotovorum * and *fungi Rhizoctonia solani, Fusarium solani* and *Alternaria dauci*	100 mg L^−1^	Inhibits the growth of all tested pathogens	[23]
SiO_2_-Ag composites	*Xanthomonas oryzae* >pv. *oryzae*	50, 100 and 200 μg mL^−1^	Displays antibacterial activity against the tested pathogen	[136]
Si	* Puccinia melanocephala *	400, 1200 mg L^−1^	Reduces disease in sugarcane and induces resistance	[137]
Si-NPs	* Fusarium oxysporum * and *Aspergillus niger*	5, 10, 15 kg ha^−1^	Reduces the growth of pathogens	[138]
Si	*Hemileia vastatrix*	0.24 and 0.30 mg kg^−1^	Inhibits infection of fungus *Hemileia vastatrix* and urediniospore germination	[28]
Si	* Colletotrichum sublineolum *	2 mmol L^−1^	Reduces growth by around 20%, acervuli found smaller in size	[139]
Si	* Podosphaera pannosa *	1 mg mL^−1^	Reduces disease severity by 46% and induces phenolic acid formation	[123]
Si	*Fusarium sulphureum*	100 and 200 mM	Decreases pathogen growth and reduces disease	[140]
SiO_2_	*Sclerosporagraminicola*	5, 10, 15 mM	Inhibits the growth of the fungal pathogen	[141]

## Data Availability

Not Applicable.
